# Sustainable Infrastructure: Recycled Concrete Aggregates for Cycle Paths

**DOI:** 10.3390/ma18010131

**Published:** 2024-12-31

**Authors:** Piotr Konca, Iwona Szer, Jacek Szer, Damian Obidowski, Dariusz Gawin, Przemysław Wiśniewski, Bartłomiej Wiśniewski, Krzysztof Jóźwik

**Affiliations:** 1 Department of Building Materials Physics and Sustainable Design, Lodz University of Technology, Av. Politechniki 6, 90-924 Lodz, Poland; iwona.szer@p.lodz.pl (I.S.); jacek.szer@p.lodz.pl (J.S.); dariusz.gawin@p.lodz.pl (D.G.); 2 Institute of Turbomachinery, Lodz University of Technology, Zeromskiego 116 Str., 90-924 Lodz, Poland; damian.obidowski@p.lodz.pl (D.O.); krzysztof.jozwik@p.lodz.pl (K.J.); 3 Zakład Remontowo Drogowy, Piotrkowska 276 Str., 90-361 Lodz, Poland; pw@zrd-wisniewska.pl (P.W.); bw@zrd-wisniewska.pl (B.W.)

**Keywords:** recycled concrete aggregate (RCA), sustainable construction, concrete design and tests, compressive strength tests, frost resistance, concrete with recycled concrete aggregate

## Abstract

The application of recycled concrete aggregates (RCAs) has become increasingly popular for different types of structures, as presented in several studies. However, depending on the type of structure and the region, RCAs might have different properties. This study aims to investigate the application of RCAs of different origins for substructure layers of the cycle paths located in Central Europe, which was not analysed previously. Recycled aggregates from an airport, road overpass, and building demolition were tested according to European standards and used to produce concretes, in which compressive strength, density, water absorption, and frost resistance were tested. After 28 days, RCA concrete had compressive strengths from 5.9 to 17.3 MPa and frost resistance ratios close to 1.0. The concrete parameters indicate that RCAs might be used for the construction of cycle path substructural layers with the appropriate class of cement and W/C ratio. To meet the requirements of EN 12390-3 to achieve class C8/10, RCA concrete with CEM II B/V 32.5 should be used with a W/C ratio of 1. To meet the requirements of D-04.05.01v02, RCA concrete with CEM II B/V 32.5 and a W/C ratio smaller than 1.50 should be used. Applying recycled RCAs in various structures helps protect natural resources by reusing materials. However, the variability in RCA properties requires testing to guarantee quality.

## 1. Introduction

In recent times, there has been a notable increase in the popularity of the bicycle as a means of transportation. Cycling routes have now become a standard feature of transport infrastructure design. A considerable number of material and technological solutions fail to meet the expectations of users and investors. In major urban centres, cycling is emerging as a viable alternative to public transportation and personal vehicles. The fundamental criteria for the design of routes and paths are safety, durability, and user comfort. The materials employed should ensure low rolling resistance, adequate roughness, and stability. In countries with well-developed road infrastructure, the construction of cycle paths utilises mineral–asphalt mixtures or cement concrete. The production of concrete, which will form the layers of trails and paths, requires the use of aggregates and cement, among other materials.

In the contemporary era, a noticeable shift towards reducing the detrimental effects of human activity on the natural environment is becoming evident across a multitude of sectors. This phenomenon extends beyond the domain of fossil fuels and encompasses a diverse array of fields. There is a growing trend among businesses and local authorities to focus their activities on energy saving, greenhouse gas emission reduction, and raw material reuse. It is the responsibility of all engineers at every stage of a project to consider the impact of their work on natural assets and to ensure that sustainability and effective management, including the minimisation of construction waste, are at the forefront of their considerations.

It is anticipated that global material resource use will reach approximately 180 billion tonnes by 2050, with high-income countries currently consuming ten times more per person than low-income countries and the planetary boundaries being exceeded [1]. It is not possible to effectively mitigate environmental impacts, including climate change and pollution, without reducing the level of resource use. Indeed, the magnitude of final waste and emissions released to the environment is determined by the level of resource use [1]. It is imperative that economic activity and human well-being are decoupled from resource use in order to achieve the Sustainable Development Goals [2] for all. In order to achieve effective decoupling, it is necessary to transform today’s linear material flows into circular ones. This can be achieved through a combination of intelligent infrastructure and product design, standardisation, reuse, recycling, and remanufacturing.

Sustainable development is a key United Nations (UN) concept, reflecting a holistic approach to addressing global challenges while ensuring that the needs of the present generation are met without compromising the ability of future generations to meet their needs [2]. The key defining document of the concept is the 2030 Agenda, which was adopted by all UN member states in 2015. The main goals in it are the 17 Sustainable Development Goals (SDGs) [2]. Waste management, closed-loop circulation of materials, and raw materials and climate protection are part of Goal 12, ensuring sustainable consumption and production patterns, and Goal 13, taking urgent action to combat climate change and its impacts. The whole concept aims to ensure comprehensive development that takes into account economic, social, and environmental aspects in order to create a fairer and more sustainable world.

As Fridolin et al. [3] observed, the utilisation of substantial quantities of construction materials and aggregates for the production of cement and concrete increased considerably in European countries from the mid-20th century to the first decade of the 21st century. The estimated annual EU consumption of natural aggregates, based on the average between 2012 and 2016, is approximately 2105 Mt. Mineral construction materials represent the largest raw material flow in the EU economy [4]. Reducing energy consumption and CO_2_ emissions in the concrete industry is a significant challenge nowadays.

Oikonomou [5] stated that one of the environmental issues associated with construction is that it results in the exploitation of 50% of the raw materials from nature, the consumption of 40% of the total energy and the production of 50% of the total waste. The extensive utilisation of recycled concrete aggregates (RCAs) can assist in mitigating the environmental impact of construction activities by facilitating the reuse of waste materials and the prevention of further extraction of natural aggregates (NAs).

One potential solution is the utilisation of Type 2 additives in cement and recycled aggregates (RCAs) in concrete. The construction industry is responsible for generating considerable quantities of waste, largely due to the demolition and refurbishment of buildings, as well as the production of concrete products. The extraction and processing of the raw materials required for concrete production has a considerable environmental impact. Each new concrete construction contributes to the generation of concrete rubble waste, which will require management in the future.

It can be posited that construction and demolition waste can be considered a suitable building material and that RCAs can be identified as raw materials for economic construction in the future. It is, therefore, possible to envisage a scenario in which construction and demolition waste is extensively used in the construction industry. This would entail collating existing knowledge, conducting further research, and creating practical guidelines that are acceptable to the industry at large [6]. The concept of reusing demolition concrete is not a novel one. In the United Kingdom, for instance, as much as 30% of the total aggregate supply is composed of recycled or secondary-use aggregates. These recycled aggregates are defined as resulting from the processing of inorganic materials that were previously utilised in construction, such as construction and demolition waste. It is asserted that recycled aggregates are employed as roadstone.

The utilisation of recycled aggregates offers a number of benefits, including a reduction in the consumption of natural resources, cost savings in concrete production, the availability and recycling of materials, a decrease in the amount of energy consumed and carbon dioxide produced in the process, and, thus, the protection of the environment [7]. Conversely, the primary disadvantages associated with the utilisation of this particular material are the discrepancies in its physical and heterogeneity, the presence of impurities, the diminished strength and adhesion in concrete, and the potential risk of reduced durability in the structure [8].

Recycled concrete aggregate (RCA)-based concrete is becoming increasingly popular in the construction industry, but its long-term performance compared to traditional concrete can vary depending on a number of factors, such as the quality of the raw materials, the recycling processes, and the concrete mixes used [9,10]. Several comparative studies can be found in the literature, e.g., [9,10], suggesting that properly designed RCA-based concrete can achieve satisfactory mechanical properties and durability and can also be lower than traditional concrete.

The literature dedicated to the study of mechanical properties [11,12] indicates the possibility of using RCAs to produce not only roads [13] but also high-performance concrete [14]. Tayeha et al. [14] suggested that RCAs can be used as both fine and coarse aggregates with high replacement ratios. On the basis of a literature review, they declared that it is even possible to obtain high-performance concrete (HPC) with total replacement.

Limitations on the use of recycled concrete aggregates (RCAs) are due to both the properties of RCAs and the performance requirements of such concrete applications.

RCAs typically have lower strength and higher porosity and water absorption compared to natural aggregates. The presence of adhering mortar can lead to reduced bond strength between aggregates and cement paste, and increases in the water/cement ratio potentially compromise the strength and durability of concrete. For high-performance concrete, this is important because the concrete must have high density, high strength, and durability. The quality of RCAs can vary significantly depending on the source material (e.g., old concrete). The variability in aggregate properties makes it difficult to achieve constant performance in high-load applications. High-performance concrete must perform well under difficult environmental conditions (e.g., freeze–thaw cycles, sulphate attack). For applications exposed to aggressive environments, the use of RCAs must be carefully considered and may require additional treatment or admixtures to increase durability. The long-term performance of RCAs in high-load concrete applications is not as well-documented as for natural aggregates, especially under extreme conditions. The long-term effects on shrinkage, creep, and crack development may be more significant compared to natural aggregates.

RCAs can be used in high-performance or high-load-bearing concrete applications, but their limitations—particularly with regard to strength, durability, and consistency—require careful consideration and adaptation of mix design.

In the paper application of RCAs for cycle paths, the substrate was only considered. There are studies where the material is used for high-performance or high-load-bearing concrete applications [14,15], but in such a case, much wider and more profound studies, including material long-term durability, are required.

In order to evaluate and contrast the environmental impact of concrete incorporating recycled aggregate, a considerable number of researchers have employed the methodology of life cycle assessment (LCA). However, the application of LCA in RCAs is not without its challenges, as there is still no standardised approach to this topic [16].

A number of researchers have conducted life cycle assessment (LCA) analyses for recycled concrete, with studies referenced in [17,18,19], for instance. It is not possible to draw general conclusions about the environmental performance of recycled concrete aggregates (RCAs) in comparison to natural aggregates (NAs) due to the multitude of parameters that can influence the results. Each case must therefore be considered separately. However, it can be concluded that the recycling of concrete can bring environmental benefits, dependent on the ratio of the distances travelled by RCAs and NAs. The location of the recycling plant is therefore crucial. It is important to note that construction aggregates are a low-value product, exhibiting considerable sensitivity to the distance transported. Each application in construction requires a specific product specification. In light of the aforementioned considerations, it is evident that the utilisation of crushed or milled concrete on the same or proximate construction site represents a clear advantage. In their research, Ohemeng and Ekolu concluded that the reuse of recycled concrete aggregates (RCAs) is economically and environmentally beneficial, as RCA production is less expensive than that of natural aggregates [20]. They also demonstrate that the environmental benefit of producing one tonne of recycled concrete aggregates was approximately 97% higher than that of natural aggregates.

The principal aim of this study was to ascertain the viability of utilising concrete derived from the demolition of diverse concrete structures for the substructure of cycle paths. There are many studies in the literature on the properties of RCA, concretes with these aggregates [21], and various additives to improve their properties [22]. This has been extensively described, e.g., by Nwakaire et al. [21]. However, in different regions of the world, the construction materials used may have different properties. Consequently, as noted by other authors [21], RCAs are also characterised by a large variation in parameters. It is, therefore, necessary to examine as many RCAs as possible from different regions of the world and different sources in order to be able to increase their use at a later stage. For example, in eastern and central Europe, in temperate, warm transitional climates, frost resistance and water absorption are of key importance in addition to durability. In the present study, the application of RCAs of different origins to be applied for cycle road substructure layers is examined. Due to the impact of the distance between the sites from which the RCAs are taken, the available sources of RCAs are considered. In particular, the demolition of existing objects in the close surroundings of a construction site is the best solution from the environmental point of view.

This paper includes an experimental study of RCAs from different sources, including an airport apron, a road overpass, and a building located in Central Europe. Section 2 presents compositions of different concrete mixtures with RCAs or a combination of RCAs and natural aggregates. The applied testing methods are described for the following RCA features: grain size distribution, dust content, grain density, water absorption, acid-soluble sulphates, frost resistance of the aggregate fraction 8/16 mm, content of recycled coarse aggregate components, and crush resistance. For RCA concretes, we describe the following features: water absorption, compressive strength, compressive strength after frost resistance cycles, and frost resistance. Section 3 contains the results of the performed tests of the RCAs and RCA concretes.

The methods for obtaining optimal pavements using recycled materials for cycle paths require further research, which are planned in the future.

An LCA analysis is not presented in this article, but such an exercise is planned for the future, taking into account energy consumption and emissions from processing and transport. This requires a lot of detailed data, which are not available for the considered case.

### Current Use of Recycled Aggregates

Dixit et al. [23] indicated that 49% of raw stone, gravel, and sand, 25% of virgin wood, and 16% of water are consumed by the construction sector. The substantial demand for concrete in the construction sector gives rise to a correspondingly significant demand for aggregates. In 2015, the global construction market was approximately 48.3 billion tonnes in volume [24]. In order to achieve sustainable civil engineering, recycled aggregates (RAs) have emerged as a potential solution to the shortage of raw materials, with the expectation that they will contribute to the conversion of construction waste into resources. Consequently, RAs have been employed as an ingredient in the production of concrete. Over the past few decades, extensive research has confirmed that recycled aggregates (RAs) can be used as a substitute for natural aggregates (NAs) [6,25]. Managing variability in recycled aggregate properties requires an integrated approach that combines technology, quality management, and materials knowledge. Applying these strategies to the management of recycled aggregates will lead to more stable and predictable production results, which is crucial on an industrial scale.

The growing consumption of natural aggregates in the construction industry has led to a necessity for their replacement with aggregates derived from alternative raw materials. In accordance with the recommendations set forth by the RILEM TC 121-DRG TF1 [26], recycled aggregates derived from demolition are classified into three categories:

- Type I—aggregate derived solely from brick rubble;
- Type II—aggregate derived solely from concrete rubble;
- Type III—mixed aggregate consisting of at least 80% natural aggregate and a maximum of 10% Type I aggregate.

The European standard EN 12620 [27] provides a classification system for recycled coarse aggregates (Rc—concrete and concrete elements, Ru—unbound aggregates, natural stones, Rb—ceramics, silicates, and aerated concrete, Ra—bituminous materials) based on the fraction content and origin of the aggregate. A summary of the conclusions drawn from studies on recycled aggregate-based concretes conducted globally in recent years can be found in reference [28]. In particular, the potential for partial and total replacement of coarse aggregate in concrete mixes with recycled aggregate was investigated. Concrete is a composite material, and its properties and performance depend on both the aggregate and cement matrix.

The higher water absorption rates of recycled concrete aggregates (RCAs) can lead to some durability problems in concrete. Recycled aggregates have a higher porosity compared to natural aggregates. Higher porosity can lead to higher water absorption, which in turn affects the mechanical properties of the concrete and its durability. High water absorption can cause greater concrete shrinkage, leading to cracking and reduced durability [29]. Water that is absorbed by RCAs can also contribute to the alkali–silica reaction (ASR), which can result in further damage to the concrete. Durability issues can lead to long-term degradation of the concrete, which affects its service life and functionality [8].

The standardised approach to the durability of concrete has changed little over the past decades. The European standard EN 206+A2:2021-08 [30] contains criteria for frost resistance in various environments (minimum 50 years). This standard is intended for use in different countries with different climatic conditions and different regional traditions and experiences in the use of concrete components. Similarly, the American standard ACI 318 [31] adopts Exposure Categories and Classes for different corrosive conditions. In China, GB (Guobiao), GB 50010-2010 [32]—“Code for Design of Concrete Structures” standard is mainly used for the design of concrete, indicating the environmental categories of the concrete structure for different environmental conditions.

For frost resistance, compressive strength, the w/c ratio, air content, and limits on cementitious materials are key parameters. There is also a new concept of equivalent performance characteristics (ECPC) corresponding to regional requirements. Satisfactory concrete performance is acceptable under local operating conditions. The greatest possibilities in this respect are offered by the equivalent performance concepts [33].

In Polish conditions, one of the principal suitability criteria is the frost resistance of recycled concrete aggregates. The issue of designing and assessing the frost resistance of concrete intended for use in road structures is discussed in depth in reference [34]. The suitability of recycled concrete aggregate for use in unbound layers and cement-bound improved base layers was analysed in [35]. In view of the potential disadvantages of using RCA aggregates over NA, namely, reduced strength, adhesion problems, heterogeneities and contaminants, the direction taken was to use a relatively safe application, namely, sub-base for cycle paths.

## 2. Materials and Methods

This research was conducted in two stages. In the first stage, recycled aggregates and natural aggregates were studied. The second stage involved the design, construction, and testing of concretes using these aggregates. Aggregate recycling is a process that allows for building materials to be reused, helping to protect the environment and reduce waste. There are several methods of obtaining recycled aggregates, which include crushing, sorting, and segregating possibly chemical contaminations that allow building materials to be broken down into their components. On the other hand, the milling of road surface layers is a key process in road upgrading and maintenance, which involves the removal of damaged concrete pavement. Milling methods can be adapted depending on the condition of the road and project specifications. Complete milling, when the entire pavement layer is removed, is used when the road is severely damaged. Partial milling, when only the top layer of the pavement is removed, is used when the layers below are to be preserved. Spot milling is the removal of small areas of pavement that are damaged. Layer milling allows several pavement layers to be removed at once, depending on their conditions. RCA obtained by partial milling were used in this study.

### 2.1. Testing of Recycled Concrete Aggregates

The initial phase of this study included the analysis of 12 recycled aggregates sourced from three distinct locations: an airport apron, a road overpass, and concrete waste resulting from a building demolition. The sampling methods employed for the testing of aggregates were in accordance with the standards set by the European Standard EN 932-1 [36], and the individual subsamples were reduced to laboratory size in accordance with the European Standard EN 932-2 [37]. The testing of aggregates was conducted in accordance with the guidelines set forth in the Technical Specification for the Execution and Acceptance of Construction Work D-04.05.01, “Improved base of cement-bound mix”, developed by the Polish General Directorate for National Roads and Highways [38], as well as the established standards for the evaluation of individual aggregate properties.

**Figure 1:**
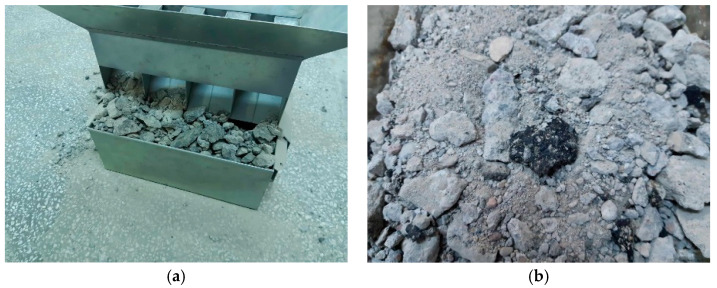
Recycled concrete aggregates: (**a**) preparation for testing; (**b**) example of a sample with visible contamination.

Prior to testing, the samples of the destructive material were properly prepared in order that they could be accurately measured in both qualitative and quantitative terms. Some of the samples contained impurities in the form of asphalt residues. Figure 1 illustrates samples of recycled aggregate prepared for testing, as well as a representative sample displaying visible contamination.

Following the removal of impurities and fragments exceeding 63 mm in size, a series of aggregate determinations were conducted, including the measurement of grain size [39], the observation of the shape of coarse aggregate (≥4 mm) separated from continuous aggregate [40], and the determination of dust content in the aggregate [39]. The remaining tests included grain density and absorbability [41], acid-soluble sulphate content and total sulphur content [42], the frost resistance of the aggregates (reference fraction for testing #8/16 mm) [43], recycled coarse aggregate component content [44], and crushing resistance [45].

To facilitate a comparative analysis of the parameters associated with the recycled aggregates, tests were conducted on three natural aggregates: coarse (8/16 gravel), designated N2; medium (2/8 gravel), designated N1; and fine (0/1 sand), designated N3. In the second stage, these aggregates served as fractions for grain size improvement.

Grain size tests were conducted utilising a MATEST SIEVE SHAKER laboratory sifter, equipped with a series of sieves with mesh side sizes ranging from 31.5 mm to 0.063 mm, including 31.5 mm, 16 mm, 8 mm, 4 mm, 2 mm, 1 mm, 0.5 mm, 0.25 mm, 0.125 mm, and 0.063 mm.

Based on the results of the initial testing phase, three categories of recycled aggregates were selected for the second stage of this study, classified according to their grain size and density. These categories were designated Z1, Z2, and Z3. It was observed that the recycled aggregates exhibited a need for improvement in grain size, necessitating the incorporation of an additional natural aggregate to enhance grain size.

### 2.2. Designing Concrete with Recycled Aggregates

The cements available on the market and the recycled aggregates, which were divided into three groups according to their grain size and density, were employed in the tests.

Two types of cement, which are typically available on the construction products market, were employed in the creation of the concrete. The cements employed in the preparation of the concrete mixture were as follows: CEM II B/V 32.5R and CEM I 42.5R cements were produced by the GÓRAŻDŻE HEIDELBERGCEMENT GROUP Cement Works. The initial Portland–fly ash cement is employed in the manufacture of concretes falling within the C 8/10 to C 30/37 classification, while the second Portland cement is utilised in the production of concretes encompassing the C 8/10 to C 40/50 and above range.

The concretes with recycled aggregates were prepared using pure water and a plasticiser *(CHRYSO^®^ Fluid CE 40).* This plasticising admixture is produced on the basis of modified polynaphthalenes. *CHRYSO^®^Fluid CE 40* has a strong dispersing action towards dusty fractions (cement, mineral additives), which enables the production of plastic and liquid mixtures with a low w/c ratio. The plasticiser increases the cohesiveness of the concrete mixture, thereby reducing the phenomenon of bleeding and segregation of concrete.

The general characteristics of the plasticiser are as follows:

- Density: 1186 ± 0.03 kg/dm^3^;
- pH: 6 ± 1;
- Chloride ion content: ≤0.1%;
- Alkali content (calculated as Na_2_O): ≤7.0%.

The mean dosage of plasticiser was 1.0% by weight of cement, and this was employed for all concrete types.

A multitude of concrete types were produced. The individual concrete mixtures were formulated in a manner that ensured the ratio of mixing water to cement remained constant, with a water-to-cement ratio (w/c) ranging between 1.00 and 1.55 for the various cements employed. Conversely, the quantity of slurry was measured to achieve the desired consistency (V1 or V3) [46], with V1 being the preferred option.

The quantity of aggregate present in a single cubic metre of concrete for the samples utilising the Z1 aggregate ranged from 1499 kg/m^3^ to 1854 kg/m^3^, while those employing the Z2 aggregate exhibited a range of 1621 kg/m^3^ to 1900 kg/m^3^, and those utilising the Z3 aggregate demonstrated a range of 1732 kg/m^3^ to 1938 kg/m^3^.

### 2.3. Testing Concrete with Recycled Concrete Aggregate

The compressive strength of the concrete series was determined after 28 days using cubic specimens with dimensions of 100 × 100 × 100 mm. The test was conducted in accordance with the specifications set forth in EN 12390-3 [47]. The loading methods and loading rates indicated in the test standard were used. A constant loading rate within the range of 0.6–0.2 (N/mm^2^/s) was chosen. After applying an initial load of approximately 30% of the failure load, the specimen was loaded continuously. The load was increased at the selected constant loading rate of ±10% until no greater loading was possible. The specimens were subjected to a compressive load until failure in a compression testing machine in accordance with the requirements of EN 12390-4 [48]. The maximum load that the specimen was able to withstand was determined, and the compressive strength of the concrete was subsequently calculated.

The concrete samples were subjected to a series of tests, including assessments of density and water absorption [49], compressive strength after 28 days [47], and frost resistance [39]. Samples for the determination of the frost resistance index [38] were stored for 28 days at room temperature with protection against drying out (in a chamber with a humidity level of 95% to 100%). Subsequently, the specimens were completely immersed in water for a period of 24 h and subjected to a series of freeze–thaw cycles over the following 14 days. A single freeze–thaw cycle entailed the freezing of the sample at a temperature of −23 ± 2 °C for a period of eight hours, followed by a defrosting process in water at a temperature of +18 ± 2 °C for a duration of 16 h.

The compressive strength was evaluated using a MATEST 3000 kN apparatus with *SERVO PLUS* evolution equipment manufacturer Matest Italy. During the frost resistance test, the samples were stored in a *TOROPOL K-015* climate chamber manufacturer TOROPOL Sp. z o.o. Poland. The concrete mixtures were prepared using mechanical mixing with a *PEMAT ZYKLOS* mixer Manufacturer Pemat Mischtechnik GmbH Germany.

## 3. Results and Discussion

This section provides a description of the experimental results, their interpretation, and the experimental conclusions that can be drawn.

### 3.1. Recycled Aggregates

**Figure 2:**
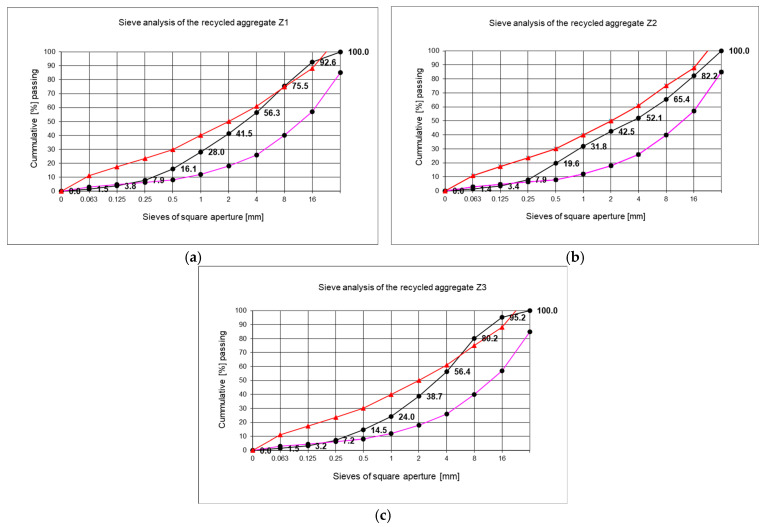
The sieve analysis of a series of samples of recycled aggregates: (**a**) Z1; (**b**) Z2; (**c**) Z3. Red line: upper limit, pink line: lower limit, black line: sieve experiment results.

The grain size range up to 31.5 mm was considered in this paper (see Table 1) because larger aggregates are not suitable for application in substructure layers of cycle paths. The results of the grain size test enabled the weight percentage of each fraction in the test sample to be determined. Figure 2 presents a graphical representation of the outcomes of the three aggregate groups (Z1, Z2, Z3) obtained through sieving with the sieves, along with the associated limit curves [38].

Figure 2 presents a graphical representation of the results obtained through sieving with the sieves and the associated limit curves [38].

The RCAs used for RCA concrete production and their tests were selected mainly on the basis of their grain size distributions, crash resistance, and frost resistance. The full results of the tests performed for the RCAs used in further concrete testing are presented in Table 1.

Table 1 presents the fundamental characteristics of the three categories of recycled aggregates, including grain size, dust content of continuous aggregate, grain density, water absorption, acid-soluble sulphates, frost resistance, content of recycled coarse aggregate components, and crush resistance.

The recycled aggregates exhibited a high content of fine fractions, as indicated by a high sand point. Concurrently, these aggregates exhibited elevated water absorption (exceeding 10% by weight), which could potentially precipitate an augmented water demand of the concrete mixture during the design phase of concretes.

The acid-soluble sulphate content was found to be at a low level, with no instances of a concentration exceeding 0.8%. Similarly, the total sulphur content was found to be below 1% in all the aggregates.

The design of concretes with adequate frost resistance and low recycled aggregate resistance may necessitate the use of admixtures to enhance this parameter or the incorporation of mixes with a low water/cement ratio.

The crush resistance (or crush factor) of recycled aggregates is several times lower than that of natural aggregates. The majority of recycled aggregates are derived from plain concrete (compressive strength up to a maximum of 60 N/mm^2^), whereas natural aggregates are derived from rocks with significantly higher strengths. The physical, chemical, and mechanical properties of recycled and natural aggregates were employed in the design of concrete mixtures.

### 3.2. Tests on Concrete Made with Recycled Aggregates

The concrete with which the tract and pathway layers will be constructed necessitated the utilisation of two distinct types of aggregate. Recycled aggregate is defined as mineral and cementitious material that has been crushed to a fine powder and bound together by a cementitious binder. This material can be produced by milling the road surface layers at ambient temperature or by crushing lumps from the demolition of old pavement or concrete structures in a crusher. It is necessary that the waste material has a grain size of up to 31.5 mm, where up to 10% oversize is permitted. In order to ensure the suitability of the mixture, it is essential that the grain size falls within the range defined by the limit curves, which represent the optimal grain size. Accordingly, the waste material resulting from the grinding of the existing pavement or concrete construction should be re-graded with aggregate. In addition, natural aggregate is employed for the re-grading of the rubble. The materials used for this purpose must comply with the requirements set forth in EN 12620+A1:2010 [27], entitled “Aggregates for concrete”. This standard encompasses aggregates with a density exceeding 2000 kg/m^3^, encompassing natural, artificial, and recycled aggregates for concrete.

Furthermore, the tested recycled aggregates also necessitated an enhancement in grain size.

Two further types of natural aggregate were employed in order to enhance this quality of the recycled aggregates. The aggregates in question were N1 (2/8 gravel) and N3 (0/1 sand). The proportions of the individual aggregates in the recycled aggregate mixture were determined by sieve analysis and were as follows:

- Aggregate Z—50%;
- Natural aggregate N1 2/8—35%;
- Natural aggregate N3 0/1—15% by weight.

**Figure 3:**
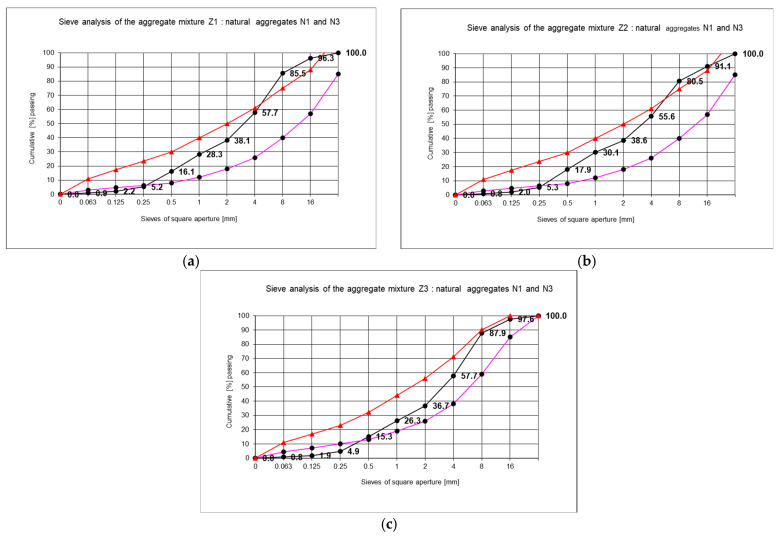
The sieve analysis of a series of samples of recycled aggregates: (**a**) recycled Z1 and natural N1 and N3; (**b**) recycled Z2 and natural N1 and N3; (**c**) recycled Z3 and natural N1 and N3. Red line: upper limit, pink line: lower limit, black line: sieve experiment results.

It should be noted that each case of recycled aggregate requires a separate composition design in order to achieve a suitable grain size curve. Figure 3 shows an example of screening curves for a mixture of Z1, Z2, and Z3 aggregates with mineral aggregates.

A multitude of concrete formulations were developed. In accordance with the findings presented in this article, sample concrete compositions were selected, comprising three types of recycled aggregates and two types of cements. The proposed concrete compositions utilise both recycled aggregates exclusively (designated BZ2-3, BZ1-11, BZ2-15, BZ3-21, and BZ3-25) and a combination of recycled and natural aggregates (designated BZ1-9). Table 2 illustrates the concrete compositions with varying proportions of ingredients, which can be employed as a foundation for the development of concrete mixtures incorporating recycled aggregates.

In the subsequent phase of the investigation, the fundamental properties of the resulting concretes were evaluated. The data obtained from the concrete tests are presented in Table 3.

The compressive strength of the proposed concretes after 28 days ranged from 5.9 MPa to 17.3 MPa. Two types of cement with different c/w ratios and three different recycled aggregates were used. This resulted in different concrete characteristics, which allow such concrete mixtures to be used in numerous projects. The compressive strength of these concretes after freezing and thawing cycles ranged from 5.8 MPa to 17.8 MPa. Therefore, the frost resistance index was approximately 1. The lowest strengths were observed in concretes with recycled aggregates and CEM II B/V 32.5 cement at a W/C ratio of 1.29. The highest strengths were observed in concretes with a mixture of recycled and natural aggregates, using CEM I 42.5R cement at a W/C ratio of 1.

**Figure 4:**
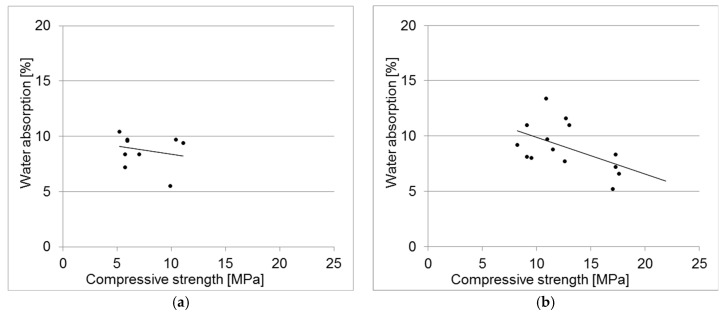
Compressive strength of concrete samples with cement, as a function of water absorption: (**a**) CEM II B/V; (**b**) CEM I 42.5R.

Figure 4 illustrates the compressive strength as a function of water absorption for all concrete samples tested. Figure 4a depicts the results for concretes using CEM II B/V 32.5R cement, while Figure 4b shows the results for those using CEM I 42.5R cement.

**Figure 5:**
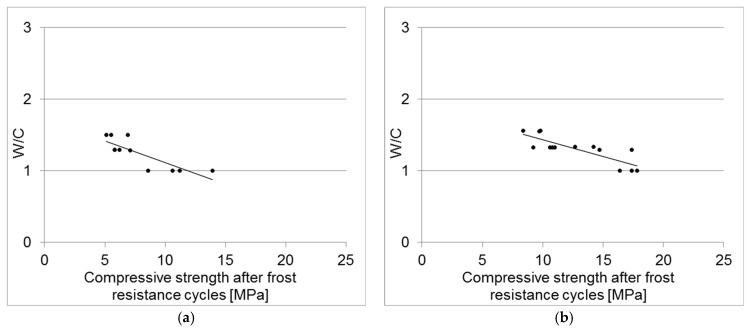
Compressive strength of concrete samples after freeze–thaw cycles, as a function of the W/C ratio, for samples made with either (**a**) CEM II B/V cement or (**b**) CEM I 42.5R cement.

Figure 5 illustrates the compressive strength after freeze–thaw cycles as a function of the water/cement ratio (W/C) for individual concrete samples. The results are presented separately for concretes containing CEM II B/V 32.5R cement and CEM I 42.5R cement.

A review of the data reveals a clear trend: as the water/cement ratio (W/C) decreases, there is an increase in both compressive strength and compressive strength after freeze–thaw cycles.

The standard that describes the requirements for concrete is EN 206+A2:2021-08, “Concrete—Specification, performance, production and conformity” [30]. This standard applies to concrete used for in situ construction, precast structures, and structural precast products used in buildings and structures. The lowest class defined in EN 206 is class C 8/10, and the standard also sets out the criteria for conformity in terms of compressive strength. The compressive strength of the concrete is assessed on samples tested on the 28th day of curing. In order to qualify for the lowest class C8/10, the average strength of the concrete, determined on cubic specimens, must be a minimum of 14 N/mm^2^. Concretes with recycled aggregates or mixtures of recycled and natural aggregates on CEM I 42.5 R cement can meet this requirement.

EN 206+A2:2021-08 [30] regulates requirements for concrete, including the use of recycled aggregates (RCAs). The latest version adds rules for their use. They can be used if their suitability is determined at the application site. The standard does not specify recommendations for recycled fine aggregate. The standard limits their use to exposure classes X0, XC1-XC4, XF1, XA1, and XD1. However, it is insufficient to meet the challenges of using RCAs. The standard does not take into account the specificity and quality of these aggregates, which can affect the durability and strength of concrete. The EN 206+A2:2021-08 [30] standard provides a proper basis for traditional concrete, but when using recycled aggregates, it is worth verifying their adequacy for specific conditions and design requirements, as well as conducting additional testing to ensure the required safety and durability of the structure.

Please refer to “Application ranges for mixtures bound with hydraulic binders for pavement construction layers and frost course”, in D-04.05.01v02 [38] for further details. The lowest class defined in this document is class C 1.5/2 (with an upper limit of 6 MPa), and the highest class is C8/10 (with an upper limit of 20 MPa). Concretes with recycled aggregates or mixtures of recycled aggregates and natural aggregates on CEM II B/V 32.5R cement or CEM I 42.5 R cement can meet the specified requirements.

The effect of concrete mix consistency on strength was also examined. The consistency of the concrete mix was altered from low-plastic V1 to plastic V3, which required an increase in the amount of cement in 1 m^3^ of concrete. However, this resulted in no change in strength at a constant W/C ratio.

Furthermore, the impact of a plasticiser, at a rate of 1% by weight of cement, was examined in the context of a low-plastic V1 concrete mix. The plasticiser used did not have a notable effect on the strengths and cement quantities in the working recipe.

## 4. Conclusions

This article presents the findings of a series of tests conducted on concrete aggregates sourced from the demolition of various structures, including an airport slab, a road overpass, and a building. Based on these results, concrete compositions were selected for use in the construction of the structural layers of cycle paths. The proposed concrete compositions included recycled concrete aggregates with and without natural aggregates. The following observations were made as a result of this study:

- The recycled concrete aggregates were found to have a high fine content and high water absorption, with an average of 14.9%.
- The compressive strengths of the concretes obtained ranged from 5.9 MPa to 17.3 MPa, depending on the type of cement and the W/C ratio. By selecting the appropriate W/C ratio and cement grade, it is possible to achieve concrete strengths across a described spectrum. To achieve concrete class C8/10, the use of CEM I 42.5 R cement with recycled aggregates or a combination of recycled and natural aggregates is recommended.
- The frost resistance factor results for all tested concretes were positive and significantly higher than the 0.6 or 0.7 values typically required for cycle path construction layers.
- The addition of natural aggregates had no significant effect on the final strength of the recycled aggregates tested at the same W/C ratio.
- Any change to the concrete recipe, including the cement manufacturer, type of cement, and aggregates or their parameters, requires a revision of the working recipe and laboratory testing of the basic properties of the concrete in each case.
- The use of recycled concrete aggregates with an appropriate concrete recipe can, therefore, be employed for the construction layers of cycle paths, providing an alternative to natural aggregates. The reuse of construction materials from the demolition of airports, road overpasses, and buildings will contribute significantly to the protection of the natural environment.

The use of recycled concrete from different sources means that the characteristics of the material are different each time. This is one of the disadvantages of using this type of material. The authors recommend that, in any case, when using demolition concrete, tests should be carried out on each batch of material in order to segregate it appropriately and, at the same time, eliminate contamination. Generalising test results to different climatic and geographic regions is often complicated and depends on several factors. Research results may be closely related to specific local conditions, the RCA source location, and the type of construction from which the RCA material is derived. If testing is performed on a small sample or has a specific scope, the results might not be representative of the larger population. Climatic and ecological conditions may change over time, which also affects the generalisability of results. In some cases, results might be generalisable if the regions studied have similar climatic characteristics. Each specific case should be approached with caution and take into account local conditions and requirements.

## Figures and Tables

**Table 1:** Properties of aggregates used in sub-base and improved sub-base layers from cement-bound mixtures.

Sample		Z1	Z2	Z3
Type of Parameter	Parameter	Aggregate Properties		
Grain size	Cat.	GA90	GA90	GA90
	d/D	0/16	0/31.5	0/16
Dust content of aggregate	[%]	1.5	1.4	1.5
Grain density	ρ_a_	2.39 Mg/m^3^	2.37 Mg/m^3^	2.50 Mg/m^3^
	ρ_rd_	1.76 Mg/m^3^	1.92 Mg/m^3^	1.84 Mg/m^3^
	ρ_ssd_	2.02 Mg/m^3^	2.11 Mg/m^3^	2.10 Mg/m^3^
	ρ_b_	1.32 Mg/m^3^	1.52 Mg/m^3^	1.31 Mg/m^3^
Water absorption	[%]	15.1%	15.5%	14.2%
Acid-soluble sulphates	Cat.	AS0.8	AS0.8	AS0.8
Frost resistance of aggregate fraction 8/16 mm (category)	Cat.	F10	F4	F25
Content of recycled coarse aggregate components	Rc	100.0%	99.8%	98.0%
Crush resistance	Index	21	27	19

**Table 2:** Selected concrete compositions employed in this study at varying w/c ratios and with two distinct cement types.

Concrete Sample Name		BZ2-3	BZ1-9	BZ1-11	BZ2-15	BZ3-21	BZ3-25
Cement type		CEM II B/V 32.5	CEM I 42.5R	CEM II B/V 32.5	CEM I 42.5R	CEM II B/V 32.5	CEM I 42.5R
Portion of cement	[kg/m^3^]	186	191	183	147	160.7	152.8
Portion of water	[kg/m^3^]	186	191	236	197	240.9	237.7
Aggregate Z	[kg/m^3^]	1791	926	1683	1797	1766.1	1780.6
Agr. N1 2/8 mm	[kg/m^3^]	0	648	0	0	0	0
Agr. N3 0/1 mm	[kg/m^3^]	0	278	0	0	0	0

**Table 3:** The test results for hardened concrete at different w/c ratios and two types of cement.

Concrete Sample Name		BZ2-3	BZ1-9	BZ1-11	BZ2-15	BZ3-21	BZ3-25
Cement type	[-]	CEM II B/V 32.5	CEM I 42.5R	CEM II B/V 32.5	CEM I 42.5R	CEM II B/V 32.5	CEM I 42.5R
W/C ratio	[-]	1.00	1.00	1.29	1.33	1.50	1.56
Concrete density	[kg/m^3^]	1980	2030	2000	2000	1960	1980
Water absorption	[%]	9.4	8.3	9.6	9.7	8.4	8.0
Compressive strength	[MPa]	11.1	17.3	5.9	11.0	7.0	9.5
Compressive strength after frost resistance cycles	[MPa]	10.6	17.8	5.8	10.6	6.9	9.7
Frost resistance ratio	[-]	1.0	1.0	1.0	1.0	1.0	1.0

## Data Availability

For reasons of corporate confidentiality, we cannot grant access to the underlying data or the results of the experiment during the life of this project. Such data can only be made available upon reasonable request after written consent from the entrepreneur.
